# Low-grade, systemic inflammation and the risk of perioperative neurocognitive disorders in an observational study of older adults

**DOI:** 10.1038/s41598-025-31986-z

**Published:** 2025-12-20

**Authors:** Insa Feinkohl, Daniel Hadzidiakos, Thomas B. Dschietzig, Jürgen Janke, Maria Heinrich, Arjen J.C. Slooter, Claudia Spies, Georg Winterer, Tobias Pischon

**Affiliations:** 1 Epidemiology Research Group, Faculty of Health Sciences Brandenburg, University of Potsdam, Potsdam, Germany; 2 Molecular Epidemiology Research Group, Max-Delbrueck-Center for Molecular Medicine in the Helmholtz Association (MDC), Berlin, Germany; 3 Charité – Universitätsmedizin Berlin, corporate member of Freie Universität Berlin and Humboldt-Universität zu Berlin, Berlin, Germany; 4 Immundiagnostik AG, Berlin, Germany; 5 MHB Medizinische Hochschule Brandenburg, Neuruppin, Germany; 6 Max-Delbrueck-Center for Molecular Medicine in the Helmholtz Association (MDC), Biobank Technology Platform, Berlin, Germany; 7 Berlin Institute of Health at Charité – Universitätsmedizin Berlin, Berlin, Germany; 8 Department of Intensive Care Medicine and UMC Utrecht Brain Center, University Medical Center Utrecht, Utrecht University, Utrecht, Netherlands; 9 Department of Psychiatry, University Medical Center Groningen, Groningen, the Netherlands; 10 Research School of Behavioral and Cognitive Neuroscience (BCN), University of Groningen, Groningen, the Netherlands; 11 PI Health Solutions GmbH, Berlin, Germany

**Keywords:** Ageing, Inflammation, Neuroepidemiology, Postoperative delirium, Postoperative cognitive dysfunction, Inflammation, Neuroimmunology, Biomarkers, Neurology, Risk factors

## Abstract

Aims The neuroinflammatory response to surgery may contribute to the pathogenesis of post-operative delirium (POD) and cognitive dysfunction (POCD), but whether inflammation before surgery enhances the risk of developing these conditions is unclear. Here, we investigate the relationship between preoperative levels of inflammation markers and the risk of POD/POCD. Methods 697 surgical patients aged ≥ 65 years were recruited 2014–2017 in Utrecht, the Netherlands and Berlin, Germany into the BioCog study. C-reactive protein (CRP), S100A12, interleukin-6 (IL-6) and IL-18 were measured immediately before surgery. POD was assessed twice daily up to 7 days/hospital discharge. POCD was determined from neuropsychological testing before surgery and 3 months thereafter. Multiple logistic regression analyses were run adjusted for age, sex, surgery site, and BMI. Results. 140 out of 697 patients (20.1%) developed POD during 7 days/by discharge; 50 out of 469 patients (10.9%) attending the 3-month follow-up developed POCD. CRP ≥ 10 mg/L was found in 149 patients and was not associated with POD/POCD. Among patients with CRP < 10 mg/L, higher S100A12 and higher CRP concentrations were each associated with higher POD risk (OR per SD higher concentrations, S100A12, 1.26, 95% CI 1.03, 1.54; CRP,1.42, 95% CI 1.15, 1.76). Higher S100A12 was associated with higher POCD risk (OR 1.40 per SD, 95% CI 1.04, 1.88). No associations were found for IL-6 and IL-18. Of note, only the result on CRP and POD survived Bonferroni correction with cut-off p < 0.006. Conclusion Low-grade inflammation may influence individual vulnerability to cognitive complications of surgery. Our results warrant further examination. Trial registration NCT02265263.

**Supplementary Information:**

The online version contains supplementary material available at 10.1038/s41598-025-31986-z.

Post-operative delirium (POD) and cognitive dysfunction (POCD) are common complications after surgery and anesthesia in older persons. POD is characterized by acute inattention and other cognitive deficits due to a medical condition after surgery, and can affect ≥ 20% of patients^1^. POCD is characterized by a decline in cognitive performance following surgery; prevalence reports vary greatly across populations and time since surgery. Although POD and POCD are distinct conditions, POD may increase later POCD risk^2^. POD and POCD are becoming increasingly relevant because of the ageing population and the increased application of surgical and anesthesiologic procedures. Both POD and POCD impair quality of life^3^ and (particularly POD) may increase the risk of dementia^1,4^.

Studies suggest that the neuroinflammatory response to surgery plays a critical role in the pathogenesis of POD and POCD^5,6^ and that anti-inflammatory drugs administered before surgery could ameliorate patients’ prognosis accordingly^7,8^. Interleukin-6 (IL-6) and interleukin-18 (IL-18) are important cytokines involved in immune activation and inflammation. IL-6 is mostly secreted by T cells and monocytes/macrophages; it promotes haematopoiesis and B cell proliferation and activation, and is a major inducer of hepatic secretion of acute-phase proteins, including C-reactive protein (CRP)^9^ as well as the calgranulin S100A12^10^. IL-18 is mostly secreted by monocytes/macrophages in many anatomical districts including the CNS and increases in most inflammatory conditions, in autoimmunity and with age^11^. IL-18 is one of the most important activators of type 1 immune responses in synergy with IL-12, by activating Th1 lymphocytes and inducing the production of high levels of IFN-γ^12^. Among its inflammatory effects, IL-18 also induces the release of acute-phase proteins^9^ and IL-6^13^. Further, CRP may induce IL-18 release^14^. S100A12, or calgranulin C, is a calcium-binding protein mainly produced by monocytes/macrophages and neutrophils; it acts as alarmin and as anti-infective agent through its ion binding capacity^15^.

Case-control studies have frequently shown that patients who develop POD or POCD have higher levels of inflammatory markers compared to unimpaired controls. For instance, elevated S100A12 and CRP have been reported in patients with POD or POCD^16,17^. However, prospective cohort studies that address whether these markers measured *before surgery* are associated with the development of these conditions could prove particularly useful in the context of risk stratification and identification of pathophysiological processes. One also needs to distinguish between the presence of clinically relevant elevations of inflammatory markers (as is often seen for example in case of acute infections) and a steady-state chronic low-grade subclinical inflammation caused by smoking, metabolic factors or ageing. Each, clinically relevant inflammation on the one hand and chronic low-grade subclinical inflammation on the other, is likely to have a different relationship with the cognitive vulnerability to surgery; yet previous studies did not apply this type of distinction. Therefore, in this cohort of older surgical patients, we determined the risk of POD and POCD associated with (i) preoperative CRP concentrations indicative of clinically relevant inflammation (CRP ≥ 10 mg/L) and (ii) preoperative low-grade inflammation (operationalized as the relative concentration of CRP, S100A12, IL-6, and IL-18 within the CRP < 10 mg/L group). The CRP ≥ 10 mg/L cut-point was selected based on its use in clinical practice in accordance with AHA^18^ and in research^19^. We hypothesized that patients with clinically relevant inflammation as well as those with relatively higher low-grade inflammation in the subclinical range were each at increased risk of developing POD and POCD.

## Materials & methods

### Study design

Our analysis was based on the Biomarker Development for Postoperative Cognitive Impairment in the Elderly (BioCog) study^20^, a multi-centre cohort of 933 patients aged ≥ 65 years who were scheduled for elective surgery in Berlin, Germany, and Utrecht, the Netherlands, during 2014–2017. Exclusion criteria were, among others, a Mini Mental State Examination score < 24; for a full list of inclusion and exclusion criteria, please see^21^. The BioCog study was registered under ClinicalTrials.gov (NCT02265263) on 2014-09-23 with Professor Claudia Spies as responsible party.

### Baseline clinical examination

Body mass index (BMI) was derived from measured height and weight. History of diabetes, hypertension, coronary heart disease (CHD), stroke, transient ischemic attack (TIA) and medication use were determined from a combination of self-report and local hospital records. Fasting blood samples were collected in supine position before surgery. Routine parameters were measured at laboratories adjacent to the hospitals. Serum and plasma samples were additionally frozen at −80˚C and shipped to a centralized biobank, where they were thawed once for aliquoting and were then re-frozen for later extraction from the biobank and measurement of inflammatory markers. Scores ≥ 5 on the German and Dutch versions of the 15-item Geriatric Depression Scale (range 0 to 15) indicated probable depression. Premorbid IQ around age 30 was estimated using the German version of the Mill-Hill Vocabulary scale in berlin participants, and the Dutch Adult Reading Test in subjects from Utrecht, which were converted to a single IQ scale.

### Surgery and anesthesia

The main inclusion criterion besides patients’ age was the expected duration of elective surgery ≥ 60 min to ensure some degree of homogeneity among the study sample. Furthermore, no restrictions were made regarding the type of surgery or discipline, so patients scheduled for diverse surgical procedures were included such as orthopedic, general, cardiac, neurosurgery, urological, gynecological and otorhinolaryngological surgery.

Anesthetic techniques were applied according to the respective standard operating procedures of the study centers for the surgical procedures and included general anesthesia (inhalational or total intravenous, respectively), regional anesthesia (i.e. neuraxial or peripheral) or combined anesthesia.

### Post-operative delirium

POD was defined according to Diagnostic and Statistical Manual of Mental Disorders (DSM-5) criteria. Patients were screened for POD by fully trained clinical staff daily after surgery at 8 am and 7 pm (± 1 h). Patients were considered as having POD if any one of the following criteria were met at any point between surgery and post-operative day 7 (or discharge): 1) ≥ 2 cumulative points on the Nursing Delirium Screening Scale (Nu-DESC); 2) positive Confusion Assessment Method (CAM) score or positive CAM for the Intensive Care Unit (CAM-ICU); and/or 3) patient chart review that shows descriptions of delirium (e.g., confused, agitated, drowsy, disorientated, received antipsychotics as treatment for delirium).

### Post-operative cognitive dysfunction

Six age-sensitive neuropsychological tests assessing various age-sensitive cognitive domains were administered by trained staff in a quiet room once during the days before surgery (baseline), once at 7 days/discharge and once 3 months after surgery (Paired Associates Learning; Verbal Recognition Memory; Spatial Span; Grooved Pegboard; Simple Reaction Time; Trail-Making Test-B). Full details can be found elsewhere^21^. POCD was defined from the change in cognitive test scores between baseline and 3-months relative to age-matched, non-surgical controls also recruited locally at the Berlin and Utrecht study centers as described previously^22^. We applied the ISPOCD criteria which are based on reliable change indices to these scores to define POCD^23^.

### Measurement of preoperative inflammatory markers

All inflammatory markers were measured from blood samples stored at the centralized biobank. Serum and plasma samples were extracted from the biobank and shipped to an analysis lab for this purpose. IL-6 and IL-18 were each analyzed in a single batch; CRP and S100A12 were measured in two batches for logistical reasons. CRP, IL-6 and IL-18 were measured in plasma. S100A12 was measured in serum using a commercially available sandwich ELISA (Immundiagnostik AG, Bensheim, Germany) with inter-assay coefficient of variation (CV) < 12%. CRP was measured using a commercially available sandwich ELISA (Immundiagnostik AG) with inter-assay CV < 10%. IL-18 was measured using the ProcartaPlex Multiplex Immunoassay kit (cat. PPX-04-MXPRKHD, Thermo Fisher Scientific Inc.; Carlsbad, CA, USA) with inter-assay CV < 14%. IL-6 was measured using a commercially available ELISA kit (cat. RD194015200R; BioVendor – Laboratorní medicína a.s., Brno, Czech Republic) with inter-assay CV < 8%. The cut-point CRP ≥ 10 mg/L was pre-specified prior to our analyses to indicate acute clinically relevant inflammation and was used to run the main analyses separately in patients with and those without acute inflammation^19^.

### Datasets for analysis and handling of missing data

**Fig. 1:**
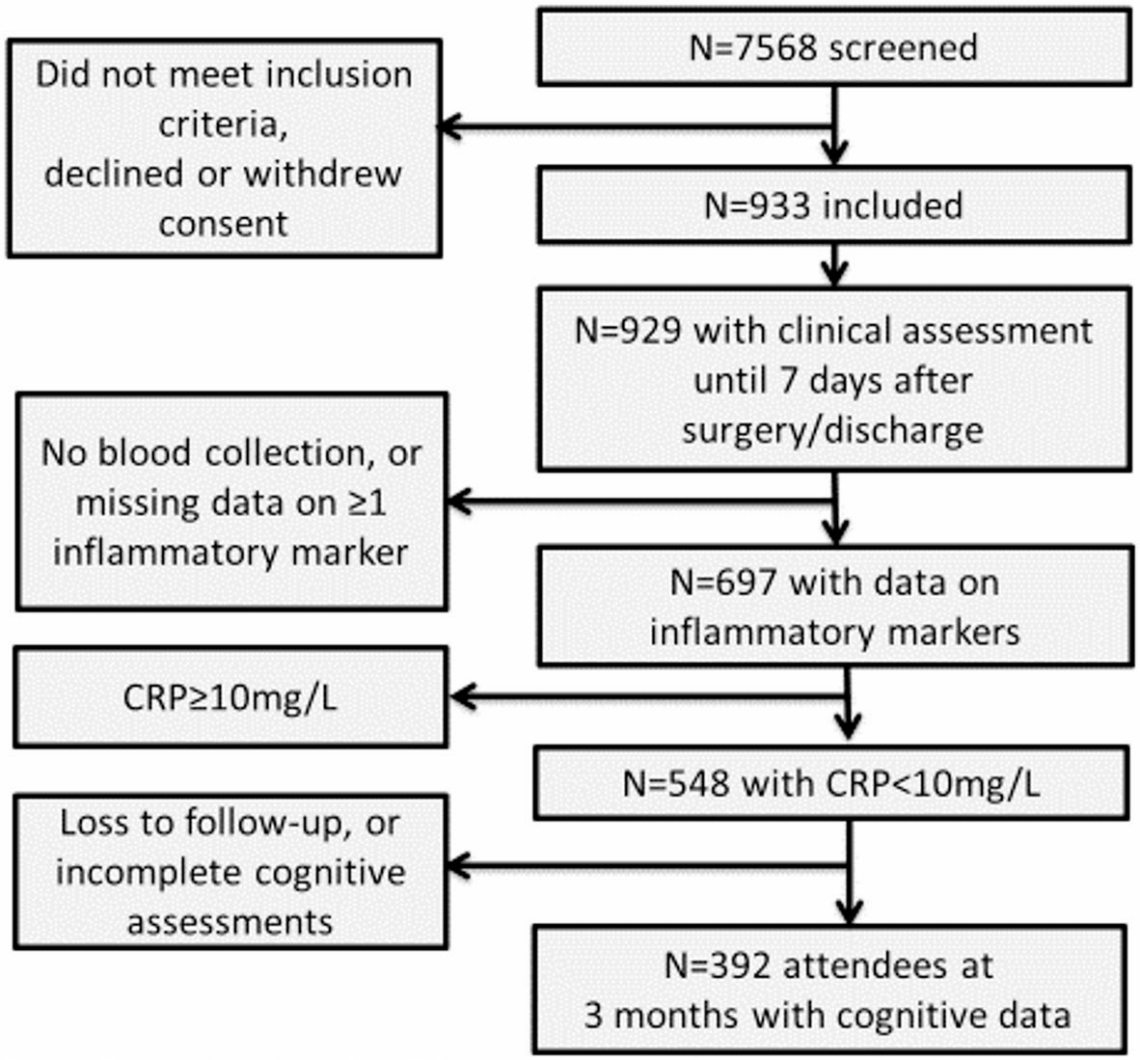
Flow chart.

Of the initial *N* = 933 cohort, 697 patients with complete data on inflammatory markers and POD were included (Supplementary Table S1; Fig. 1). For patients with missing data on diabetes, hypertension, CHD, TIA or stroke, the respective condition was assumed to be absent (of *n* = 697, diabetes, *n* = 11, 1.6%; hypertension, *n* = 12, 1.7%; CHD, *n* = 19, 2.7%; TIA, *n* = 19, 2.7%; stroke = 15, 2.2%). Missing data on pre-morbid IQ (*n* = 121, 17.4%) were replaced by median IQ (111), anesthesia duration (*n* = 13, 1.9%) by median duration (201 min), and surgical site (*n* = 14, 2.90%), by the most common site (peripheral surgery). Missing depression scores were replaced by the median (*n* = 113, GDS = 1.1). For 188 patients (27.0%), IL-6 concentration was below the detection limit and was replaced by the lowest measured value in the sample (0.037 pg/mL). Results for IL-6 presented in this manuscript were similar in terms of effect sizes and statistical non-significance when these patients were excluded (data not shown).

### Statistical analysis

Across the total *N* = 697 sample, multiple logistic regression analyses examined the association of preoperative CRP ≥ 10 mg/L (yes/no) with risk of POD and POCD, respectively. All subsequent analyses of the inflammatory markers and risk of POD/POCD were performed on the subsample of 548 patients with CRP < 10 mg/L as the group without presence of clinically relevant inflammation (for analyses of total sample, see Supplemental Data). Spearman rank correlation analyses explored the associations among the four inflammatory markers. Principal component analysis (PCA) was applied to the four individual inflammatory markers (CRP, S100A12, IL-6, IL-18) to derive a hypothesized composite latent factor (‘inflammation factor’) that was immune to the measurement error affecting the four individual inflammatory markers. Patients were divided into quartiles based on the distribution of each inflammatory marker. The covariate structure according to inflammatory quartiles was assessed using chi^2^ tests and analyses of variance (ANOVA; see Supplemental Data). We assessed the association of inflammatory markers and the ‘inflammation factor’ (as quartiles and continuous) with POD/POCD risk using multiple logistic regression analyses with hierarchical statistical adjustment for potential confounders (model 1) and mediators (model 2) through entering these factors into the respective model, as outlined in detail below.

In model 1, we adjusted for age, sex, surgery site and BMI as potential confounding factors, as well as for fasting status and analysis batch of the lab analyses. In model 2, we additionally entered diabetes, hypertension, CHD, TIA and stroke, because these are generally accompanied by low-grade inflammation and may in fact result from inflammatory processes, and additionally predispose to POD/POCD (e.g^24)^. Thus, they are candidate mediating factors which would lead to statistically non-significant results in this step. In model 2, we also adjusted for anesthesia duration as potential mediator related directly to surgery. To limit the total number of statistical analyses, post-hoc adjustment steps were limited to statistically significant associations in model 2. Specifically, we adjusted the respective model 2 for pre-morbid IQ and depression scores. These covariates were selected, because a lower pre-morbid IQ and depression have been associated with cognition after surgery^25^ as well as with inflammation^26^, and thus could act as confounders (for instance, leading both to inflammation and POD/POCD). For POCD, analyses were additionally adjusted for POD as a potential mediator. None of the findings reported here were changed in these post-hoc steps (data not shown). IBM SPSS software was used for analysis and a p-value < 0.05 in two-tailed analyses was considered statistically significant.

## Results

### Sample characteristics

Patients in the full analysis sample (*N* = 697) had a mean age of 72 (standard deviation, SD 5) years, and 57.8% were male (Table S1). 149 patients (21.4%) had preoperative CRP ≥ 10 mg/L and were more likely to be male, to have a history of cancer, were more likely to receive antibacterial medication, had a higher BMI, higher levels of S100A12, IL-6 and IL-18, and had a longer duration of anesthesia compared with the 548 patients with CRP < 10 mg/L (Table 1). POD was detected in 140 (20.1%) patients and 50 (10.1% of 469 attendees of follow-up) had POCD at 3 months.

**Table 1:** Baseline characteristics and surgery-related factors according to preoperative CRP concentrations.

Characteristics	Total analysis sample *N* = 697	CRP ≥ 10 mg/L *n* = 149	CRP < 10 mg/L *n* = 548	*p*-value^a^
Male, n (%)	402 (57.7)	73 (49.0)	329 (60.0)	0.02
Age, years, mean ± SD	72.3 ± 5.0	72.6 ± 5.1	72.2 ± 4.9	0.32
Study center				0.22
Utrecht, n (%)	152 (21.8)	27 (18.1)	125 (22.8)	
Berlin, n (%)	545 (78.2)	122 (81.9)	423 (77.2)	
Surgery				0.04
Intracranial, n (%)	9 (1.3)	1 (0.7)	8 (1.5)	
Thoracic/abdominal/pelvic, n (%)	306 (43.9)	79 (53.0)	227 (41.4)	
Peripheral, n (%)	382 (54.8)	69 (46.3)	313 (57.1)	
Duration of anaesthesia (min), median (interquartile range)	201 (121, 310)	233 (160–397)	186 (144–289)	< 0.001
Depression (GDS ≥ 5), n (%)	62 (8.9)	16 (10.7)	46 (8.4)	0.37
Present or past history of tumour, leukemia or lymphoma^b^, n (%)	229 (34.7)	72 (51.1)	160 (30.8)	< 0.001
History of coronary artery disease, n (%)	131 (18.8)	33 (22.1)	98 (17.9)	0.24
History of stroke, n (%)	35 (5.0)	9 (6.0)	26 (4.7)	0.52
History of transient ischemic attack, n (%)	20 (4.2)	8 (5.4)	21 (3.8)	0.41
History of diabetes, n (%)	159 (22.8)	46 (30.9)	113 (20.6)	0.008
History of hypertension, n (%)	437 (62.7)	103 (69.1)	334 (60.9)	0.07
Body mass index (BMI; kg/m^2^), mean ± SD	27.2 ± 4.7	28.1 ± 6.1	26.9 ± 4.2	0.03
Antibacterial medication^c^, n (%)	18 (2.8)	9 (6.5)	9 (1.8)	0.003
NSAIDs^c^, n (%)	76 (12.0)	12 (8.7)	64 (12.9)	0.17
Steroids^c^, n (%)	36 (5.7)	11 (7.9)	25 (5.1)	0.20
Not Fasting, n (%)	9 (1.3)	3 (2.0)	6 (1.1)	0.38
MMSE, median (interquartile range)	29 (28, 30)	29 (28–30)	29 (28–30)	0.60
Pre-morbid IQ, mean ± SD	112.1 ± 14.5	111.6 ± 12.3	112.0 ± 13.4	0.70
S100A12 (ng/mL), median (interquartile range)	108.2 (64.2–203.7)	196.2 (98.6–346.1)	95.3 (58.7–170.5)	< 0.001
CRP (mg/L), median (interquartile range)	3.5 (1.4–8.4)	19.7 (13.1–36.3)	2.4 (1.1–5.0)	< 0.001
IL-6 (pg/mL), median (interquartile range)	2.0 (0.4–5.1)	7.4 (3.2–16.0)	1.5 (0.4–3.4)	< 0.001
IL-18 (pg/mL), median (interquartile range)	36.4 (26.2–50.4)	42.2 (29.7–57.7)	35.6 (25.7–49.1)	0.001

Data shown following imputation of missing data. CRP, C-reactive protein; GDS, Geriatric Depression Scale; IL-6, interleukin 6; IL-18, interleukin-18; MMSE, Mini Mental State Examination; NSAIDS, non-steroidal anti-inflammatory drugs. ^a^for difference between groups with versus without acute infection tested using Mann-Whitney, t-test or chi^2^ test ^b^data available for 660 patients ^c^data available for 634 patients.

### Presence of preoperative CRP ≥ 10 mg/L and risk of POD or POCD

Preoperative CRP ≥ 10 mg/L was not statistically significantly associated with POD or POCD risk (model 1, for POD, OR 1.23, 95% CI 0.78, 1.93; for POCD, OR 1.71, 95% CI 0.82, 3.56).

### Associations among inflammatory markers

Covariate structures according to biomarker quartiles are presented in Tables S2, S3, S4 and S5. In the group with CRP < 10 mg/L (*n* = 548), which served as analysis sample for all subsequent analyses, CRP was positively associated with S100A12 and IL-6 (Table 2; for correlation analyses in full sample, see Table S6). We identified a single latent ‘inflammation factor’ with Eigenvalue > 1, which explained 30.95% of the total variance in inflammatory marker data (factor loadings: S100A12, 0.72; CRP, 0.60; IL-18, 0.46; IL-6, 0.39).

**Table 2:** Correlation among inflammatory markers in patients with CRP < 10 mg/L.

	S100A12	CRP	IL-6
CRP	0.18 (< 0.001)	--	
IL-6	0.05 (0.18)	0.28 (< 0.001)	--
IL-18	0.06 (0.20)	0.07 (0.11)	−0.02 (0.70)

*N* = 548. Spearman rank correlation analyses. Values are rho (p-value). CRP, C-reactive protein; IL-6, interleukin 6; IL-18, interleukin 18.

### Preoperative low-grade inflammation and risk of POD

Of the 548 patients with CRP < 10 mg/L, 103 (18.8%) developed POD. Unadjusted results in this group are visually presented in Supplemental Figures S1 and S2. After controlling for age, sex, fasting, analysis batch, surgery site and BMI (model 1), higher S100A12 concentrations, higher CRP concentrations and higher inflammation factor scores were each associated with an increased risk of POD. Thus, the OR of POD was 1.26 (95% CI 1.03, 1.54) per 1 SD higher S100A12 concentration; 1.42 (95% CI 1.15, 1.76) per 1 SD higher CRP concentration within the CRP < 10 mg/L range, and 1.32 (95% CI 1.08, 1.62) per 1 SD higher inflammation factor score (Table 3). The results remained similar when potential mediating factors were additionally controlled for in model 2 (Table 3). IL-6 and IL-18 were not associated with POD risk (Table 3). For results of the total sample, see Table S7.

**Table 3:** Adjusted odds ratio and 95% CI of postoperative delirium for quartiles of inflammatory markers, and for continuous inflammatory markers in patients with CRP < 10 mg/L.

		Quartiles of concentration					Continuously		
		I	II	III	IV	*P*_trend_	OR (95% CI) per 1 or per 10 unit(s) increment	OR (95% CI) per 1 SD increment	*P*_OR_^a^
S100A12 (ng/mL)	Model 1	1.0 (Ref)	1.09 (0.56, 2.13)	1.06 (0.55, 2.07)	1.84 (0.99, 3.44)	0.15	1.02 (1.00, 1.04)^c^	1.26 (1.03, 1.54)	0.03
	Model 2	1.0 (Ref)	1.26 (0.62, 2.56)	1.29 (0.63, 2.63)	2.49 (1.27, 4.88)	0.03	1.03 (1.01, 1.05)^c^	1.35 (1.10, 1.67)	0.004*
C-reactive protein (mg/L)	Model 1	1.0 (Ref)	0.92 (0.45, 1.90)	1.34 (0.68, 2.67)	2.53 (1.33, 4.83)	0.004*	1.15 (1.06, 1.25)^b^	1.42 (1.15, 1.76)	0.001*
	Model 2	1.0 (Ref)	0.94 (0.44, 1.99)	1.39 (0.68, 2.82)	2.56 (1.31, 4.97)	0.006	1.14 (1.05, 1.25)^b^	1.40 (1.12, 1.75)	0.003*
IL-6 (pg/mL)	Model 1	1.0 (Ref)	1.15 (0.57, 2.29)	1.44 (0.79, 2.64)	1.44 (0.79, 2.62)	0.57	0.97 (0.86, 1.09)^c^	0.93 (0.70, 1.23)	0.60
	Model 2	1.0 (Ref)	1.14 (0.56, 2.32)	1.11 (0.58, 2.13)	1.22 (0.65, 2.27)	0.94	0.97 (0.85, 1.10)^c^	0.92 (0.67, 1.25)	0.58
IL-18 (pg/mL)	Model 1	1.0 (Ref)	1.32 (0.72, 2.40)	0.52 (0.26, 1.06)	1.01 (0.96, 1.07)	0.05	0.99 (0.93 1.07)^c^	0.98 (0.76, 1.26)	0.86
	Model 2	1.0 (Ref)	1.30 (0.69, 2.43)	0.52 (0.24, 1.10)	1.33 (0.70, 2.51)	0.05	1.00 (0.94, 1.08)^c^	1.01 (0.79, 1.29)	0.93
Inflammation factor	Model 1	1.0 (Ref)	1.82 (0.90, 3.69)	1.96 (0.97, 3.96)	2.36 (1.18, 4.72)	0.11	--	1.32 (1.08, 1.62)	0.007
	Model 2	1.0 (Ref)	1.96 (0.93, 4.12)	2.24 (1.07, 4.72)	2.73 (1.30, 5.72)	0.06	--	1.37 (1.12, 1.69)	0.003*

*N* = 548. CI, confidence interval; IL-6, interleukin 6; IL-18, interleukin 18; OR, odds ratio; SD, standard deviation. 1.0 (ref) denotes the comparison category for quartile analyses.

Upper quartile cut-points : S100A12, Q1 : 63.6ng/mL, Q2 : 107.8ng/mL, Q3 : 206.4ng/mL; CRP, Q1 : 1.4 mg/L, Q2 : 3.4 mg/L, Q3 : 8.3 mg/L; IL-6, Q1 : 0.04pg/mL, Q2 : 1.99pg/mL, Q3 : 5.08pg/mL; IL-18, Q1 : 26.4pg/mL, Q2 : 36.4pg/mL, Q3 : 49.4pg/mL.

p-value for trend (two-sided) across quartiles is based on the median inflammatory marker concentrations within quartiles, used as a continuous variable and analyzed using the Wald chi^2^ statistic.

Quartiles of inflammatory markers and inflammation factor have been created on the subsample of 548 patients with CRP < 10 mg/L.

Model 1: adjusted for age, sex, fasting, analysis batch, surgery site, BMI.

Model 2: +diabetes, hypertension, CHD, TIA, stroke, anaesthesia duration.

^a^addition of quadratic term into model 2 in a separate step resulted in the following p-values for quadratic terms: S100A12, *p* = 0.34; CRP, *p* = 0.67; IL-6, *p* = 0.63; IL-18, *p* = 0.64; ‘Inflammation Factor’, *p* = 0.10.

^b^OR per 1 unit increment ^c^OR per 10 units increment.

*statistically significant at Bonferroni-corrected *p* < 0.006.

### Preoperative low-grade inflammation and risk of POCD

Among 392 patients with CRP < 10 mg/L who returned for 3-month follow-up, 37 (9.4%) had POCD at 3 months. In this group, S100A12 was associated with POCD risk. In model 1, each 1 SD increment in S100A12 was associated with a 1.37-fold increased risk of POCD (model 2, OR 1.40, 95% CI 1.04, 1.88; Table 4). The remaining inflammatory markers were not associated with POCD (Table 4). For results of the total sample, see Table S8.

**Table 4:** Adjusted odds ratio and 95% CI of postoperative cognitive dysfunction for quartiles of inflammatory markers, and for continuous inflammatory markers in patients with CRP < 10 mg/L.

		Quartiles of concentration					Continuously		
		I	II	III	IV	*P*_trend_	OR (95% CI) per 1 or per 10 unit(s) increment	OR (95% CI) per 1 SD increment	*P*_OR_^a^
S100A12 (per ng/mL)	Model 1	1.0 (Ref)	1.40 (0.50, 3.90)	0.91 (0.31, 2.68)	2.03 (0.78, 5.30)	0.34	1.03 (1.00, 1.06)^c^	1.40 (1.04, 1.88)	0.03
	Model 2	1.0 (Ref)	1.40 (0.50, 3.97)	0.92 (0.31, 2.77)	1.89 (0.72, 4.99)	0.46	1.03 (1.00, 1.05)^c^	1.37 (1.02, 1.85)	0.04
C-reactive protein (per mg/L)	Model 1	1.0 (Ref)	0.94 (0.35, 2.54)	1.11 (0.43, 2.91)	0.95 (0.33, 2.76)	0.99	1.03 (0.89, 1.19)^b^	1.07 (0.74, 1.54)	0.72
	Model 2	1.0 (Ref)	0.91 (0.33, 2.49)	1.09 (0.41, 2.89)	0.96 (0.33, 2.82)	0.99	1.03 (0.89, 1.19)^b^	1.08 (0.75, 1.56)	0.69
IL-6 (per pg/mL)	Model 1	1.0 (Ref)	0.80 (0.26, 2.48)	0.92 (0.35, 2.43)	1.44 (0.60, 3.48)	0.69	0.99 (0.88, 1.11)^c^	0.98 (0.73, 1.30)	0.99
	Model 2	1.0 (Ref)	0.80 (0.25, 2.51)	0.99 (0.37, 2.64)	1.66 (0.68, 4.07)	0.53	0.99 (0.88, 1.12)^c^	0.98 (0.74, 1.31)	0.90
IL-18 (per pg/mL)	Model 1	1.0 (Ref)	0.87 (0.34, 2.24)	0.81 (0.30, 2.19)	1.02 (0.39, 2.69)	0.96	0.93 (0.78, 1.11)^c^	0.77 (0.41, 1.46)	0.43
	Model 2	1.0 (Ref)	0.95 (0.36, 2.48)	0.76 (0.28, 2.10)	1.02 (0.38, 2.70)	0.95	0.93 (0.77, 1.11)^c^	0.77 (0.40, 1.46)	0.42
‘Inflammation factor’	Model 1	1.0 (Ref)	1.35 (0.48, 3.80)	1.29 (0.44, 3.76)	1.61 (0.57, 4.59)	0.85	--	1.15 (0.86, 1.55)	0.35
	Model 2	1.0 (Ref)	1.41 (0.49, 4.03)	1.28 (0.44, 3.77)	1.59 (0.55, 4.60)	0.86	--	1.16 (0.86, 1.57)	0.34

*N* = 392. CI, confidence interval; IL-6, interleukin 6; IL-18, interleukin 18; OR, odds ratio; SD, standard deviation. 1.0 (ref) denotes the comparison category for quartile analyses.

Upper quartile cut-points : S100A12, Q1 : 63.6ng/mL, Q2 : 107.8ng/mL, Q3 : 206.4ng/mL; CRP, Q1 : 1.4 mg/L, Q2 : 3.4 mg/L, Q3 : 8.3 mg/L; IL-6, Q1 : 0.04pg/mL, Q2 : 1.99pg/mL, Q3 : 5.08pg/mL; IL-18, Q1 : 26.4pg/mL, Q2 : 36.4pg/mL, Q3 : 49.4pg/mL.

p-value for trend (two-sided) across quartiles is based on the median inflammatory marker concentrations within quartiles, used as a continuous variable and analyzed using the Wald chi^2^ statistic.

Quartiles of inflammatory markers and inflammation factor have been created on the subsample of 548 patients with CRP < 10 mg/L.

Model 1: adjusted for age, sex, fasting, analysis batch, surgery site, BMI.

Model 2: +diabetes, hypertension, CHD, TIA, stroke, anaesthesia duratiuon.

^a^addition of quadratic term into model 2 in a separate step resulted in the following p-values for quadratic terms: S100A12, *p* = 0.38; CRP, *p* = 1.00; IL-6, *p* = 0.55; IL-18, *p* = 0.73; ‘Inflammation Factor’, *p* = 0.86.

^b^OR per 1 unit increment ^c^OR per 10 units increment.

*statistically significant at Bonferroni-corrected *p* < 0.006.

## Discussion

In this cohort of older patients undergoing elective surgery, we found that CRP concentrations indicative of clinically relevant inflammation before surgery (CRP ≥ 10 mg/L) was not associated with POD or POCD. Among patients with low-grade subclinical inflammation (CRP < 10 mg/L), higher preoperative S100A12 and CRP concentrations and a higher composite inflammation factor score were each associated with an increased POD risk, supporting the independent role of low-grade inflammation. Higher preoperative S100A12 was also associated with an increased risk of POCD at 3 months, and independently of its relationship with POD suggesting that POD did not function as a mediating factor. Effect sizes were considerable throughout and for S100A12 were similar for associations with POD and with POCD. All associations were independent of age, sex, surgery-related factors, vascular risk factors, vascular disease, pre-morbid IQ and depression. IL-6 and IL-18 were not associated with POD or POCD throughout.

Several epidemiological studies found higher levels of inflammatory markers in patients with POD or POCD as compared to controls (e.g^16)^, but whether these observations are cause or consequence of POD or POCD often remained unclear. To the best of our knowledge, S100A12 and IL-18 had never been assessed as preoperative risk factors in the context of POD/POCD, and all prior research on continuous preoperative CRP and IL-6 did not stratify according to CRP < 10 mg/L vs. CRP ≥ 10 mg/L. A meta-analysis across 9 studies of this type^27^, as well as similar studies not included in the meta-analysis^28^ or published thereafter^29–32^, consistently found an association of a higher preoperative full-range CRP with an increased POD risk. For the outcome POCD null findings on associations with preoperative full-range CRP appear more common^27,33–37^, though some investigations^17,38^ and a meta-analysis (limited to studies on total hip replacement) did find evidence in this direction^39^. Importantly, however, we cannot fully interpret the evidence from those types of studies, because a distinction between analyses of patients with CRP < 10 mg/L and those with CRP ≥ 10 mg/L is essential; variations in inflammatory markers among persons with CRP < 10 mg/L likely reflect chronic, subclinical conditions; CRP above the 10 mg/L threshold are typically seen in clinically relevant inflammatory conditions. These include acute infection, rheumatoid arthritis and cancer for instance. Here, in the first study to apply such a distinction, we have shown that CRP ≥ 10 mg/L was not a risk factor for POD or POCD, thus extending previous mixed findings from studies on lower CRP cut-points (≥ 3 mg/L or ≥ 5 mg/L)^28,36,40^. Additionally, higher S100A12 and CRP concentrations, indicative of an increased degree of low-grade subclinical inflammation, were each associated with an increased POD and, for S100A12, also with POCD risk. This suggests a detrimental role of higher concentrations within patients without clinically relevant inflammation. The pattern of results for CRP as a continuous measure among the CRP < 10 mg/L group versus categorical measure at the ≥ 10 mg/L cut-point may appear contradictory on first inspection. It is due to the fact that CRP captures different underlying processes below (low-grade subclinical inflammation) and above that threshold (clinically relevant inflammation).

We did not have reliable data on acute infection or chronic inflammatory conditions, but found a higher prevalence of a present/past history of cancer in the CRP ≥ 10 mg/L group compared with the CRP < 10 mg/L group. This could suggest systemic effects of cancer treatments or cancer itself on CRP levels. In contrast to the group with CRP ≥ 10 mg/L, variations in inflammatory markers among persons with CRP < 10 mg/L are most likely related to lifestyle, nutritional, or metabolic factors^41^ and reflect variation in chronic exposure. Further, chronic subclinical inflammation is a risk factor for cardiovascular disease. AHA and others have proposed to distinguish persons with low (CRP < 1.0 mg/L), medium (1.0-<3.0 mg/L) and high (3.0-<10 mg/L) cardiovascular risk according to CRP^18^. Interestingly, in our analysis, we found that variations in inflammatory marker levels among persons with CRP < 10 mg/L are also associated with risk of POD, suggesting that chronic low-grade inflammation may be a risk factor not only for cardiovascular disease but also for delirium after surgical interventions.

Our focus on patients with CRP < 10 mg/L may also explain the disparity between our null findings on IL-6 and a meta-analysis that had reported an association with POD^27^. Studies included in the meta-analysis had again assessed total study samples, and they mainly reported unadjusted results. Interestingly, when we repeated our analyses on the total sample, in model 1 with minimal adjustment, IL-6 quartiles were associated with POD risk (see Supplemental Data) as was IL-6 as a continuous measure in a crude model run post hoc (data not shown). These associations disappeared in model 1 (continuous analyses) and model 2 (quartile analyses) respectively. Clearly, adjustment for confounders is central to avoiding causal interpretation of associations that are in fact driven by some third factor. Results from meta-analyses pooling data from studies that did not apply statistical adjustment for confounders (e.g^27^), thus need to be interpreted with caution. At the same time, our findings on IL-6 and IL-18 may also suggest that measurement of free plasma concentrations of the biomarkers, as was the case here, may not reflect appropriately the concentrations of bio-active compounds, given that they bind to soluble receptor (sIL-6R) or to a binding protein (IL-18BP) for instance. The pleiotropic nature of IL-6 and IL-18 also makes it difficult to determine by which mechanism they act in detail on target cells further downstream the signalling pathway^42^.

The mechanisms leading to perioperative NCD are unknown, but the inflammatory response to surgical trauma is recognized as a likely contributor. Peripheral inflammation initiates neuroinflammatory responses such as an activation of glial cells and leads to increased blood-brain barrier permeability^43^. Here, we have shown that a higher level of low-grade subclinical inflammation predisposes patients to a more substantial impact of these types of responses on brain function. The association could be mediated by neurovascular damage^44^ following (potentially years of) chronic, low-grade, subclinical systemic inflammation, for instance due to metabolic dysfunction^41^. This would be consistent with metabolic dysfunction as a risk factor for POD^45,46^ and POCD^24^. For S100A12 in particular, which acts as an alarmin amplifying inflammation^15^, potential pathways linking it to neurological outcomes after surgery could involve an impact of the biomarker (for instance through adhesion to RAGE^47^ on blood-brain barrier function (which has been demonstrated for other inflammatory markers^48^. S100A12 has also been found to be upregulated after brain injury^49^ and has been implicated in AD-type pathology^50^ which itself could predispose to negative cognitive outcomes after surgery^51^.

Importantly, the associations found here were independent of all considered potential confounding factors, including pre-morbid IQ. We had reasoned that people with a lower pre-morbid IQ could be exposed to late-life inflammation^52^ as well as to an increased cognitive risk^53,54^. Because we found no evidence in favour of this pathway, our finding support a potential causal relationship of inflammation with cognitive risk after surgery.

Our findings have implications for risk stratification and for prevention of POD/POCD, because patients’ (subclinical) inflammatory status is modifiable. Numerous randomized clinical trial (RCTs) have addressed the perioperative cognitive risk following intra- or post-operative interventions^55^, which can be considered treatment rather than preventive strategy. Future RCTs should assess the effect of *preoperative* anti-inflammatory measures (e.g. drugs, dietary changes or physical activity) to reduce the systemic inflammatory burden and their potential effects on perioperative NCD. Such RCTs will help determine whether a short-term anti-inflammatory intervention before surgery can ameliorate any effects of chronic low-grade subclinical inflammation on cognitive outcomes. They should be applied in a structured program in the sense of prehabilitation with adequate time interval prior to elective surgery. In addition to interventions such as non-steroidal anti-inflammatory drugs, dietary changes or physical activity, the use of preoperative therapeutic antibodies or binding proteins could also be assessed. Intra-operative anti-inflammatory treatment has been shown to benefit patient health^56^ and so this route of investigation for pre-operative targeting of the pro-inflammatory state could prove fruitful.

Mechanistic studies could be used to approach the specific pathways underlying the present findings. For future analyses of clinically relevant inflammation in observational studies, data collection on the specific underlying causes (e.g., acute infection) as well as analyses of associations of dynamic changes in inflammatory markers as well as in neuroimaging data across surgery with cognitive outcomes should be strived for.

Our study has several strengths. We used prospectively collected data from a large international cohort that allowed stratified analyses and control over confounders. Because we enrolled patients undergoing diverse surgical procedures, our results are likely generalizable to the general older Caucasian population. At the same time, this selection can be seen as a limitation, because we do not know whether our results apply to Europeans of non-Caucasian ethnicity. One of the central aims of the BioCog study was to allow molecular genetic investigations and Caucasian ethnicity was chosen as a selection criterion to achieve homogeneity for this purpose.

Some further limitations must be considered. We applied a CRP cut-off of 10 mg/L to represent clinical inflammation based on prior literature though we accept that this cut-off is arbitrary and results will differ depending on cut-off selection. Selection bias will inevitably have been generated due to ‘healthier’ patients choosing to participate in the first place, with even ‘healthier’ patients (including cognitively healthier patients) choosing to return for 3-month follow-up^57^. As such, our lack of findings on the POCD analysis could in part be driven by the fact that sample characteristics were different to the POD analysis sample. The generalizability of our results to the general surgical population can also be expected to be limited as a result. Conditions such as cancer and medication use will have affected inflammatory marker concentrations and could also contribute to POD/POCD (thus could act as confounders) but were not considered in our analysis. We thus cannot say with certainty whether or not our findings may have been driven by unmeasured factors such as those. We used simple imputation methods to deal with missing data on covariates and must note that more complex approaches such as multiple imputation may be preferable. We used logistic regression and calculated odds ratios, which is an approximation of relative risk. We ran a large number of individual statistical tests (across 4 exposures and 2 outcomes), which will have increased the risk of type I error. In fact, Bonferroni correction of p-values results in a threshold for statistical significance of *p* < 0.006 and only our results on CRP and POD consistently survived this adjustment. Our statistically significant results on the remaining inflammatory markers should therefore be interpreted with caution. Due to loss to follow-up, results on POD cannot be strictly compared with those on POCD. Due to loss to follow-up, and due to a low incidence of POCD, statistical power in analyses of POCD was limited. This may explain why despite a substantial effect size (patients with CRP ≥ 10 mg/L at a 71% increased POCD risk), confidence intervals were large and statistical significance was not reached.

We used data on inflammatory markers from a single blood collection before surgery and from frozen bloods, but these markers remain relatively stable over time^58^. Moreover, CRP can be considered to be stable across freeze-thaw cycles. In one study assessing the difference between thawed and prefreeze baseline concentrations, these were reported to be within acceptable limits^59^. We therefore interpret these concentrations in the group with CRP < 10 mg/L as indicative of the degree of chronic low-grade inflammation. Further, we can consider patients with CRP ≥ 10 mg/L to be subjected to clinically relevant inflammation at least at that specific time point^18^.

Aside from inflammatory processes, a number of other risk factors have been proposed to be play a role in the development of POD/POCD. These include lifestyle factors such as excessive alcohol consumption, anticholinergic medication, smoking, frailty and nutritional status as well as surgery-related factors such as surgery type and anaesthesia method. It was beyond the scope of this work to fully examine the respective contributions to the associations observed here.

Our findings support the hypothesis that chronic, low-grade, subclinical inflammation contributes to an increased vulnerability to the effects of surgery on brain function. Findings are most robust for CRP and POD; as for the remaining biomarkers and POCD, results did not survive Bonferroni correction. Evaluation of the effects of anti-inflammatory drugs before surgery on postoperative cognitive risk offers a promising avenue for future research.

## Supplementary Information

Below is the link to the electronic supplementary material.

Supplementary Material 1

## Data Availability

The data presented here is not publicly available as due to the nature of this research, participants of the original study did not agree for their data to be shared publicly. Requests for data access can be sent to Claudia Spies (claudia.spies@charite.de) and may be granted in accordance with legal data protection regulations. Requests regarding analysis details may be sent to Insa Feinkohl (insa.feinkohl@uni-potsdam.de).
