# Supplementary material for: Low-grade, systemic inflammation and the risk of perioperative neurocognitive disorders in an observational study of older adults
Source: Sci Rep. 2025 Dec 20;15:44231. doi: 10.1038/s41598-025-31986-z (PMC12722205; doi:10.1038/s41598-025-31986-z)
Supplement: Supplementary file 1 — Supplementary Material 1 [file 41598_2025_31986_MOESM1_ESM.docx]

**Supplementary Data**

Table S1: Baseline sample characteristics and surgery-related factors for full cohort and analysis sample

| Characteristics | Total BioCog cohort  N=933 | Not included in analysis sample  n=236 | Analysis sample  n=697 | p-value^a^ |
| --- | --- | --- | --- | --- |
| Male, n (%) | 538 (57.7%) | 136 (57.6%) | 402 (57.7%) | 0.99 |
| Age, years, mean ± SD | 72.4 ± 5.1 | 72.7 ± 5.3 | 72.3 ± 5.0 | 0.29 |
| Study center |  |  |  | <0.001 |
| Utrecht, n (%) | 247 (26.5%) | 95 (40.3%) | 152 (21.8%) |  |
| Berlin, n (%) | 686 (74.5%) | 141 (59.7%) | 545 (78.2%) |  |
| Surgery |  |  |  | 0.16 |
| Intracranial, n (%) | 10 (1.1%) | 1 (0.4%) | 9 (1.3%) |  |
| Thoracic/abdominal/pelvic, n (%) | 397 (42.6%) | 91 (38.6%) | 306 (43.9%) |  |
| Peripheral, n (%) | 526 (56.4%) | 144 (61.0%) | 382 (54.8%) |  |
| Duration of anaesthesia (min), median (interquartile range) | 202 (124, 306) | 211 (132, 296) | 201 (121, 310) | 0.34 |
| Depression (GDS≥5), n (%) | 83 (8.9%) | 21 (8.9%) | 62 (8.9%) | 1.00 |
| History of coronary artery disease, n (%) | 182 (19.5%) | 51 (21.6%) | 131 (18.8%) | 0.35 |
| History of stroke, n (%) | 54 (5.8%) | 19 (8.1%) | 35 (5.0%) | 0.09 |
| History of transient ischemic attack, n (%) | 33 (3.5%) | 4 (1.7%) | 20 (2.9%) | 0.08 |
| History of diabetes, n (%) | 204 (21.9%) | 45 (19.1%) | 159 (22.8%) | 0.23 |
| History of hypertension, n (%) | 589 (61.3%) | 152 (64.4%) | 437 (62.7%) | 0.64 |
| Body mass index (kg/m^2^), mean ± SD | 27.1 ± 4.6 | 26.9 ± 4.5 | 27.2 ± 4.7 | 0.43 |
| Fasting, n (%) | 922 (98.8%) | 234 (99.2%) | 688 (98.7%) | 0.59 |
| MMSE, median (interquartile range) | 29 (28, 30) | 29 (28, 30) | 29 (28, 30) | 0.72 |
| Pre-morbid IQ, mean ± SD | 111.6 ± 13.4 | 110.8 ± 14.7 | 112.1 ± 14.5 | 0.25 |

Data shown following imputation of missing data. GDS, Geriatric Depression Scale; MMSE, Mini Mental State Examination ^a^for difference analysis sample (n=697) versus not included in analysis sample (n=236) using Mann-Whitney, t-tests or chi^2^ tests

Table S2. Characteristics of study participants by quartiles of S100A12 concentration

|  | S100A12 | | | | *P*-value |
| --- | --- | --- | --- | --- | --- |
|  | Quartile 1 | Quartile 2 | Quartile 3 | Quartile 4 |  |
| N | 137 | 137 | 137 | 137 |  |
| S100A12 concentration, range, ng/mL | 7 – 58 | 60 – 95 | 95 – 170 | 171 – 775 |  |
| Age, years, mean | 72.4 | 71.4 | 72.4 | 72.5 | 0.25 |
| Male sex, % | 64.2 | 67.9 | 59.1 | 48.9 | 0.009 |
| Depression, % | 8.8 | 5.8 | 10.9 | 8.0 | 0.50 |
| Body mass index, kg/m^2^, mean | 26.5 | 27.2 | 26.6 | 27.3 | 0.26 |
| Hypertension, % | 56.9 | 57.7 | 65.0 | 64.2 | 0.38 |
| Diabetes, % | 18.2 | 23.4 | 19.7 | 21.2 | 0.76 |
| Coronary heart disease, % | 20.4 | 15.3 | 19.0 | 16.8 | 0.70 |
| Stroke, % | 2.2 | 5.8 | 6.6 | 4.4 | 0.36 |
| Transient ischemic attack, % | 2.9 | 3.6 | 7.3 | 1.5 | 0.08 |
| Pre-morbid IQ, mean | 110 | 111 | 112 | 115 | 0.005 |
| POD, % | 15.3 | 16.8 | 16.8 | 26.3 | 0.08 |
| POCD^a^, % | 7.5 | 9.7 | 7.0 | 14.1 | 0.31 |

N=548. ^a^n=392

Table S3. Characteristics of study participants by quartiles of C-reactive protein concentration

|  | C-reactive protein | | | | *P*-value |
| --- | --- | --- | --- | --- | --- |
|  | Quartile 1 | Quartile 2 | Quartile 3 | Quartile 4 |  |
| N | 137 | 137 | 137 | 137 |  |
| C-reactive protein concentration, range, mg/L | 0.1 – 1-1 | 1.1 – 2.4 | 2.4 – 5.0 | 5.0 – 9.9 |  |
| Age, years, mean | 72.1 | 71.7 | 72.2 | 72.7 | 0.39 |
| Male sex, % | 67.2 | 58.4 | 59.9 | 54.7 | 0.20 |
| Depression, % | 3.6 | 8.0 | 8.8 | 13.1 | 0.05 |
| Body mass index, kg/m^2^, mean | 25.6 | 27.1 | 27.4 | 27.6 | <0.001 |
| Hypertension, % | 55.5 | 58.4 | 65.0 | 65.0 | 0.27 |
| Diabetes, % | 19.0 | 21.9 | 22.6 | 19.0 | 0.82 |
| Coronary heart disease, % | 19.0 | 19.0 | 15.3 | 18.2 | 0.84 |
| Stroke, % | 3.6 | 5.1 | 5.1 | 5.1 | 0.92 |
| Transient ischemic attack, % | 5.1 | 1.5 | 5.1 | 3.6 | 0.35 |
| Pre-morbid IQ, mean | 114 | 112 | 111 | 111 | 0.14 |
| POD, % | 13.1 | 13.1 | 18.2 | 30.7 | <0.001 |
| POCD^a^, % | 9.5 | 8.3 | 10.8 | 9.1 | 0.94 |

N=548. ^a^n=392

Table S4. Characteristics of study participants by quartiles of IL-6 concentration

|  | IL-6 | | | | *P*-value |
| --- | --- | --- | --- | --- | --- |
|  | Quartile 1 | Quartile 2 | Quartile 3 | Quartile 4 |  |
| N | 180^b^ | 94 | 137 | 137 |  |
| IL-6 concentration, range, pg//mL | 0.04 | 0.04 – 1.47 | 1.47 – 3.40 | 3.42 – 423.73 |  |
| Age, years, mean | 71.6 | 72.4 | 71.8 | 73.3 | 0.01 |
| Male sex, % | 55.0 | 64.9 | 61.3 | 62.0 | 0.37 |
| Depression, % | 4.4 | 10.6 | 10.2 | 10.2 | 0.14 |
| Body mass index, kg/m^2^, mean | 26.3 | 27.3 | 27.6 | 26.8 | 0.02 |
| Hypertension, % | 56.1 | 62.8 | 61.3 | 65.7 | 0.36 |
| Diabetes, % | 16.7 | 17.0 | 22.6 | 26.3 | 0.14 |
| Coronary heart disease, % | 10.6 | 22.3 | 19.7 | 22.6 | 0.02 |
| Stroke, % | 2.2 | 5.3 | 7.3 | 5.1 | 0.20 |
| Transient ischemic attack, % | 5.0 | 2.1 | 1.5 | 5.8 | 0.17 |
| Pre-morbid IQ, mean | 114 | 110 | 111 | 111 | 0.03 |
| POD, % | 15.0 | 18.1 | 20.4 | 22.6 | 0.35 |
| POCD^a^, % | 8.1 | 7.7 | 8.2 | 13.8 | 0.42 |

N=548. ^a^n=392

^b^quartile consists of patients with IL-6 below detection level (replaced by 0.037 for the purpose of our analysis)

Table S5. Characteristics of study participants by quartiles of IL-18 concentration

|  | IL-18 | | | | *P*-value |
| --- | --- | --- | --- | --- | --- |
|  | Quartile 1 | Quartile 2 | Quartile 3 | Quartile 4 |  |
| N | 137 | 136 | 138 | 137 |  |
| IL-18 concentration range, pg/mL | 10 – 26 | 26 – 36 | 36 – 49 | 49 – 657 |  |
| Age, years, mean | 72.5 | 72.8 | 71.3 | 72.1 | 0.08 |
| Male sex, % | 63.5 | 57.4 | 61.6 | 57.7 | 0.67 |
| Depression, % | 8.0 | 8.8 | 7.2 | 9.5 | 0.92 |
| Body mass index, kg/m^2^, mean | 26.3 | 26.9 | 27.8 | 26.6 | 0.02 |
| Hypertension, % | 62.0 | 52.2 | 62.3 | 67.2 | 0.08 |
| Diabetes, % | 16.8 | 18.4 | 25.4 | 21.9 | 0.30 |
| Coronary heart disease, % | 19.7 | 18.4 | 17.4 | 16.1 | 0.88 |
| Stroke, % | 5.1 | 4.4 | 5.8 | 3.6 | 0.86 |
| Transient ischemic attack, % | 6.6 | 3.7 | 1.4 | 3.6 | 0.18 |
| Pre-morbid IQ, mean | 111 | 112 | 112 | 114 | 0.30 |
| POD, % | 19.0 | 24.3 | 10.9 | 21.2 | 0.03 |
| POCD^a^, % | 9.8 | 9.7 | 8.2 | 10.0 | 0.97 |

N=548. ^a^n=392

Table S6. Univariate associations among inflammatory markers in total sample

|  | S100A12 | CRP | IL-6 |
| --- | --- | --- | --- |
| CRP | 0.31 (<0.001) | -- |  |
| IL-6 | 0.22 (<0.001) | 0.50 (<0.001) | -- |
| IL-18 | 0.09 (0.02) | 0.14 (<0.001) | 0.07 (0.05) |

N=697. Spearman rank correlation analyses. Values are rho (p-value). CRP, C-reactive protein; IL-6, interleukin 6; IL-18, interleukin 18

Table S7: Adjusted odds ratio and 95% CI of POD for quartiles of inflammatory markers, and for continuous inflammatory markers

|  |  | Quartiles of concentration | | | | | Continuously | | |
| --- | --- | --- | --- | --- | --- | --- | --- | --- | --- |
|  |  | I | II | III | IV | *P*_trend_ | OR (95% CI)  per 1 or per 10 unit(s) increment | OR (95% CI)  per 1 SD increment | *P*_OR_^a^ |
| S100A12  (ng/mL) | Model 1 | 1.0 (Ref) | 1.06 (0.59, 1.90) | 0.93 (0.52, 1.66) | 1.94 (1.12, 3.36) | 0.02 | 1.01 (1.00, 1.02)^c^ | 1.13 (0.95, 1.34) | 0.16^d^ |
|  | Model 2 | 1.0 (Ref) | 1.12 (0.61, 2.08) | 1.08 (0.59, 1.98) | 2.16 (1.21, 3.85) | 0.02 | 1.01 (1.00, 1.02)^c^ | 1.15 (0.97, 1.37) | 0.12^d^ |
| C-reactive protein  (mg/L) | Model 1 | 1.0 (Ref) | 0.89 (0.94, 1.64) | 1.52 (0.86, 2.67) | 1.57 (0.89, 2.78) | 0.11 | 1.01 (1.00, 1.02)^b^ | 1.18 (1.01, 1.38) | 0.04 |
|  | Model 2 | 1.0 (Ref) | 0.94 (0.50, 1.76) | 1.54 (0.86, 2.77) | 1.39 (0.77, 2.52) | 0.26 | 1.01 (1.00, 1.02)^b^ | 1.15 (0.97, 1.37) | 0.11 |
| IL-6  (pg/mL) | Model 1 | 1.0 (Ref) | 0.90 (0.49, 1.67) | 1.77 (1.02, 3.07) | 1.62 (0.94, 2.80) | 0.04 | 1.04 (0.99, 1.10)^c^ | 1.15 (0.98, 1.34) | 0.09^d^ |
|  | Model 2 | 1.0 (Ref) | 0.79 (0.42, 1.50) | 1.50 (0.85, 2.66) | 1.22 (0.69, 2.18) | 0.18 | 1.04 (0.99, 1.09)^c^ | 1.13 (0.96, 1.32) | 0.14^d^ |
| IL-18  (pg/mL) | Model 1 | 1.0 (Ref) | 0.75 (0.44, 1.29) | 0.61 (0.35, 1.06) | 1.00 (0.60, 1.66) | 0.23 | 1.01 (0.95, 1.07)^c^ | 1.03 (0.85, 1.24) | 0.77^d^ |
|  | Model 2 | 1.0 (Ref) | 0.84 (0.48, 1.47) | 0.60 (0.33, 1.08) | 1.13 (0.66, 1.92) | 0.17 | 1.02 (0.96, 1.08)^c^ | 1.05 (0.87, 1.27) | 0.58^d^ |
| ‘Inflammation factor’ | Model 1 | 1.0 (Ref) | 1.41 (0.78, 2.52) | 1.46 (0.82, 2.63) | 1.91 (1.09, 3.37) | 0.17 | -- | 1.24 (1.05, 1.47) | 0.01 |
|  | Model 2 | 1.0 (Ref) | 1.49 (0.81, 2.75) | 1.58 (0.85, 2.92) | 1.92 (1.05, 3.50) | 0.21 | -- | 1.22 (1.03, 1.46) | 0.02 |

N=697. CI, confidence interval; IL-6, interleukin 6; IL-18, interleukin 18; OR, odds ratio. 1.0 (ref) denotes the comparison category for quartile analyses.

p-value for trend (two-sided) across quartiles is based on the median inflammatory marker concentrations within quartiles, used as a continuous variable and analyzed using the Wald chi^2^ statistic.

Quartiles of inflammatory markers and inflammation factor have been created on total sample. Inflammation factor explained 35.73% of the total variance in observed inflammatory marker data (factor loadings: S100A12, 0.71; CRP, 0.68; IL-6, 0.60; IL-18, 0.33).

Model 1: adjusted for age, sex, fasting, analysis batch, surgery site, BMI

Model 2: +diabetes, hypertension, CHD, TIA, stroke, anaesthesia duratiuon

^a^addition of quadratic term into model 2 in a separate step resulted in the following p-values for quadratic terms: S100A12, p=0.045; CRP, p=0.40; IL-6, p=0.47; IL-18, p=0.60; ‘Inflammation Factor’, p=0.10.

^b^OR per 1 unit increment

^c^OR per 10 units increment

^d^interaction terms in separate post-hoc analyses, model 1: CRP*S100A12, p=0.88; CRP*IL-6, p=0.03; CRP*IL-18, p=0.61; model 2: CRP*S100A12, p=0.79; CRP*IL-6, p=0.07; CRP*IL-18, p=0.80

Table S8: Adjusted odds ratio and 95% CI of POCD for quartiles of inflammatory markers, and for continuous inflammatory markers

|  |  | Quartiles of concentration | | | | | Continuously | | |
| --- | --- | --- | --- | --- | --- | --- | --- | --- | --- |
|  |  | I | II | III | IV | *P*_trend_ | OR (95% CI)  per 1 or per 10 unit(s) increment | OR (95% CI)  per 1 SD increment | *P*_OR_^a^ |
| S100A12  (ng/mL) | Model 1 | 1.0 (Ref) | 1.20 (0.52, 2.79) | 0.87 (0.36, 2.08) | 1.16 (0.48, 2.76) | 0.89 | 1.01 (0.99, 1.03)^c^ | 1.22 (0.90, 1.66) | 0.20^d^ |
|  | Model 2 | 1.0 (Ref) | 1.21 (0.52, 2.82) | 0.88 (0.36, 2.12) | 1.12 (0.47, 2.69) | 0.98 | 1.01 (0.99, 1.03)^c^ | 1.22 (0.89, 1.67) | 0.21^d^ |
| C-reactive protein  (mg/L) | Model 1 | 1.0 (Ref) | 1.05 (0.44, 2.49) | 1.03 (0.41, 2.54) | 1.60 (0.66, 3.90) | 0.67 | 1.01 (1.00, 1.03)^b^ | 1.22 (0.92, 1.63) | 0.17 |
|  | Model 2 | 1.0 (Ref) | 1.03 (0.43, 2.46) | 1.03 (0.41, 2.56) | 1.64 (0.66, 4.04) | 0.65 | 1.01 (1.00, 1.03)^b^ | 1.23 (0.93, 1.64) | 0.15 |
| IL-6  (pg/mL) | Model 1 | 1.0 (Ref) | 1.23 (0.50, 3.03) | 1.42 (0.59, 3.37) | 1.36 (0.58, 3.22) | 0.87 | 1.00 (0.91, 1.10)^c^ | 1.01 (0.75, 1.36) | 0.93^d^ |
|  | Model 2 | 1.0 (Ref) | 1.28 (0.51, 3.21) | 1.44 (0.60, 3.47) | 1.47 (0.61, 3.54) | 0.82 | 1.00 (0.91, 1.10)^c^ | 1.00 (0.74, 1.35) | 0.99^d^ |
| IL-18  (pg/mL) | Model 1 | 1.0 (Ref) | 1.00 (0.46, 2.21) | 0.88 (0.38, 2.21) | 0.70 (0.29, 1.70) | 0.85 | 0.94 (0.81, 1.09)^c^ | 0.81 (0.49, 1.34) | 0.41^d^ |
|  | Model 2 | 1.0 (Ref) | 0.99 (0.45, 2.20) | 0.87 (0.37, 2.04) | 0.68 (0.28, 1.66) | 0.83 | 0.94 (0.80, 1.09)^c^ | 0.80 (0.48, 1.33) | 0.40^d^ |
| ‘Inflammation factor’ | Model 1 | 1.0 (Ref) | 0.93 (0.38, 2.31) | 1.05 (0.42, 2.58) | 1.79 (0.76, 4.20) | 0.39 | -- | 1.17 (0.84, 1.63) | 0.35 |
|  | Model 2 | 1.0 (Ref) | 0.91 (0.36, 2.28) | 1.05 (0.42, 2.60) | 1.76 (0.74, 4.17) | 0.41 | -- | 1.17 (0.83, 1.63) | 0.37 |

N=469. CI, confidence interval; IL-6, interleukin 6; IL-18, interleukin 18; OR, odds ratio. 1.0 (ref) denotes the comparison category for quartile analyses.

p-value for trend (two-sided) across quartiles is based on the median inflammatory marker concentrations within quartiles, used as a continuous variable and analyzed using the Wald chi^2^ statistic.

Quartiles of inflammatory markers and inflammation factor have been created on total sample. Inflammation factor explained 35.73% of the total variance in observed inflammatory marker data (factor loadings: S100A12, 0.71; CRP, 0.68; IL-6, 0.60; IL-18, 0.33).

Model 1: adjusted for age, sex, fasting, analysis batch, surgery site, BMI

Model 2: +diabetes, hypertension, CHD, TIA, stroke, anaesthesia duratiuon

^a^addition of quadratic term into model 2 in a separate step resulted in the following p-values for quadratic terms: S100A12, p=0.51; CRP, p=0.11; IL-6, p=0.28; IL-18, p=0.66; ‘Inflammation Factor’, p=0.08.

^b^OR per 1 unit increment

^c^OR per 10 units increment

^di^nteraction terms in separate post-hoc analyses, model 1: CRP*S100A12, p=0.17; CRP*IL-6, p=0.48; CRP*IL-18, p=0.88; model 2: CRP*S100A12, p=0.18; CRP*IL-6, p=0.48; CRP*IL-18, p=0.88


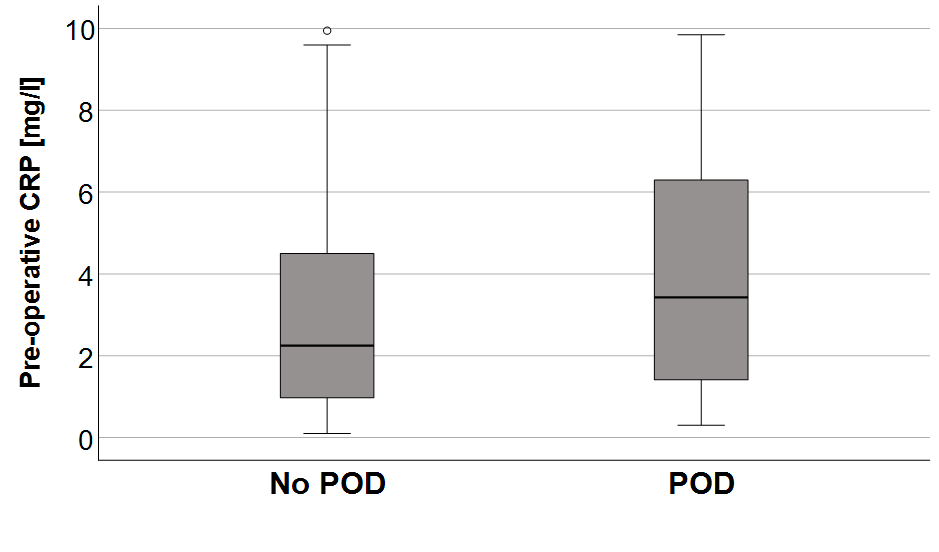


Supplemental Figure S1: Pre-operative CRP according to POD versus no POD among the CRP<10mg/l group


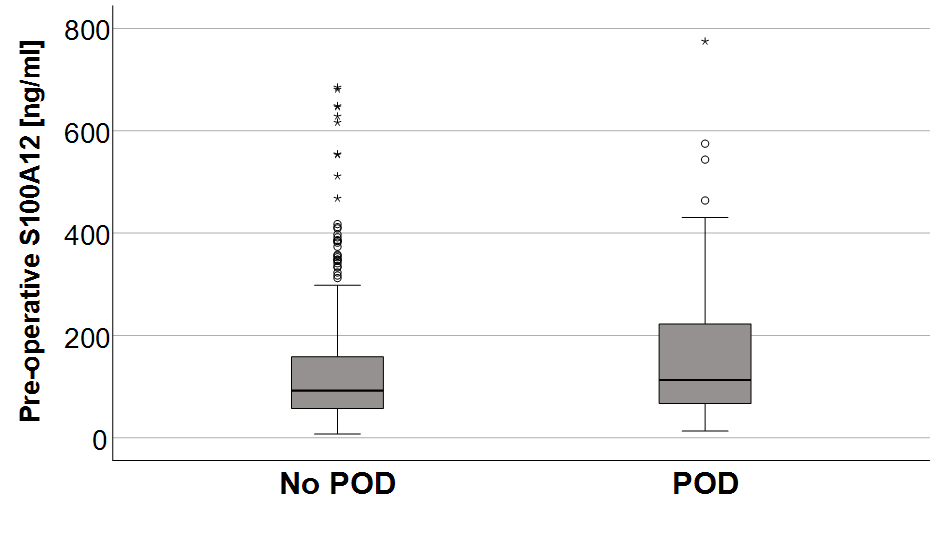


Supplemental Figure S2: Pre-operative S100A12 according to POD versus no POD among the CRP<10mg/l group
